# Six newly sequenced chloroplast genomes from prasinophyte green algae provide insights into the relationships among prasinophyte lineages and the diversity of streamlined genome architecture in picoplanktonic species

**DOI:** 10.1186/1471-2164-15-857

**Published:** 2014-10-04

**Authors:** Claude Lemieux, Christian Otis, Monique Turmel

**Affiliations:** Institut de biologie intégrative et des systèmes, Département de biochimie, de microbiologie et de bio-informatique, Université Laval, Québec, QC G1V 0A6 Canada

**Keywords:** Viridiplantae, Prasinophytes, Prasinococcales, *Picocystis*, Picoplanktonic algae, *Nephroselmis*, Plastid genome, Phylogenomics, Genome reduction, *trans*-spliced group II intron

## Abstract

**Background:**

Because they represent the earliest divergences of the Chlorophyta, the morphologically diverse unicellular green algae making up the prasinophytes hold the key to understanding the nature of the first viridiplants and the evolutionary patterns that accompanied the radiation of chlorophytes. Nuclear-encoded 18S rDNA phylogenies unveiled nine prasinophyte clades (clades I through IX) but their branching order is still uncertain. We present here the newly sequenced chloroplast genomes of *Nephroselmis astigmatica* (clade III) and of five picoplanktonic species from clade VI (*Prasinococcus* sp. CCMP 1194, Prasinophyceae sp. MBIC 106222 and *Prasinoderma coloniale*) and clade VII (*Picocystis salinarum* and Prasinophyceae sp. CCMP 1205). These chloroplast DNAs (cpDNAs) were compared with those of the six previously sampled prasinophytes (clades I, II, III and V) in order to gain information both on the relationships among prasinophyte lineages and on chloroplast genome evolution.

**Results:**

Varying from 64.3 to 85.6 kb in size and encoding 100 to 115 conserved genes, the cpDNAs of the newly investigated picoplanktonic species are substantially smaller than those observed for larger-size prasinophytes, are economically packed and contain a reduced gene content. Although the *Nephroselmis* and *Picocystis* cpDNAs feature a large inverted repeat encoding the rRNA operon, gene partitioning among the single copy regions is remarkably different. Unexpectedly, we found that all three species from clade VI (Prasinococcales) harbor chloroplast genes not previously documented for chlorophytes (*ndhJ*, *rbcR*, *rpl21*, *rps15*, *rps16* and *ycf66*) and that *Picocystis* contains a *trans*-spliced group II intron. The phylogenies inferred from cpDNA-encoded proteins are essentially congruent with 18S rDNA trees, resolving with robust support all six examined prasinophyte lineages, with the exception of the Pycnococcaceae.

**Conclusions:**

Our results underscore the high variability in genome architecture among prasinophyte lineages, highlighting the strong pressure to maintain a small and compact chloroplast genome in picoplanktonic species. The unique set of six chloroplast genes found in the Prasinococcales supports the ancestral status of this lineage within the prasinophytes. The widely diverging traits uncovered for the clade-VII members (*Picocystis* and Prasinophyceae sp. CCMP 1205) are consistent with their resolution as separate lineages in the chloroplast phylogeny.

## Background

Thriving mainly in marine environments, prasinophytes constitute an heterogeneous assemblage of unicellular green algae that occupy the earliest-diverging lineages of the Chlorophyta, i.e. the division of the Viridiplantae containing the bulk of extant green algae [1–3]. Sister to the Chlorophyta is the Streptophyta, which comprises freshwater green algae, the so-called charophytes, and all their land plant relatives. Given their basal positions, prasinophytes hold the key to understanding the nature of the last common ancestor of all green plants and the origin of the core chlorophytes (i.e. the Pedinophyceae, Chlorodendrophyceae, Trebouxiophyceae, Ulvophyceae and Chlorophyceae). The first green plants were undoubtedly unicellular algae bearing nonmineralized organic scales on their cell body and/or their flagella, because flagellated cells of many prasinophytes and streptophytes are covered by a layer of square-shaped scales [4]. Prasinophytes exhibit considerable diversity with respect to cell shape and size, flagella number and behavior, mitotic and cytokinetic mechanisms, and biochemical features such as accessory pigments and storage products [2, 5–8]. They include the smallest free-living eukaryote known to date (*Ostreococcus tauri* with an average size of 0.8 μm [9, 10]). Some prasinophytes lack flagella, others lack scales, and in some cases, both flagella and scales are absent (e.g., *Ostreococcus*).

Analyses of the nuclear-encoded small subunit (SSU) rRNA gene (18S rRNA gene) have identified nine monophyletic groups of prasinophytes, known as clades I through IX [11–14]. Most of these clades correspond to existing orders or classes; clades II, III and IV have recently been ranked at the class level (Mamiellophyceae [15], Nephroselmidophyceae [16] and Chlorodendrophyceae [17], respectively). Clades VIII and IX, the two most recently identified clades, are composed uniquely of environmental sequences [14]. The interrelationships between prasinophyte clades are still uncertain, considering that most of the internal branches separating these clades in 18S rDNA trees received weak support values. Only for the class Chlorodendrophyceae (clade IV), which is nested within the core chlorophytes, is there confidence that it represents the latest divergence in the prasinophyte radiation. Six clades (clade II, clade V (Pycnococcaceae, Pseudocourfieldiales), clade VI (Prasinococcales), and clades VII through IX) display picoplanktonic species (i.e. organisms with a diameter of less than 3 μm), two of which (clades II and V) exhibit both the coccoid (no scales nor flagella) and flagellated cell organizations. It appears therefore that small-sized prasinophytes evolved multiple times from larger ancestors, presumably because selection favored smaller cells with less requirements per cell for nitrogen, phosphorus and other elements and a reduced tendency to be captured by filter-feeding predators [18].

Comparative analysis of chloroplast genomes has been helpful to resolve various issues concerning the relationships among green algal lineages [19–24]. To date, complete chloroplast genome sequences have been described for six prasinophytes that represent four distinct clades: clade I (Pyramimonadales), *Pyramimonas parkeae* [25]; clade II, *Monomastix* sp. OKE-1 [25], *Micromonas* sp. RCC 299 [26], and *Ostreococcus tauri* [27]; clade III, *Nephroselmis olivacea* [28]; and clade V, *Pycnococcus provasolii* [25]. These genomes display considerable fluidity in overall structure, gene content, and gene order. Whereas the 200-kb *N. olivacea* genome harbors the largest gene repertoire yet reported for a chlorophyte (128 different conserved genes compared with about 138 for the deepest branching streptophyte algae) and has retained many ancestral gene clusters, the genomes from the clade-II members belonging to the Mamiellales, which are the smallest (~72 kb) and most compact chlorophyte chloroplast DNAs (cpDNAs) known to date, display a reduced set of genes (86 and 88 genes) whose order is highly scrambled. The 80-kb *Pycnococcus* and 114-kb *Monomastix* genomes (98 and 94 conserved genes, respectively) resemble their *Ostreococcus* and *Micromonas* homologs in featuring a reduced derived pattern of evolution, while the 102-kb *Pyramimonas* genome is more alike *Nephroselmis* cpDNA in harboring conserved genes unrecognized in other chlorophytes (*rpl22* and *ycf65*) as well as a DNA primase gene putatively acquired from a virus. Like most of their chlorophyte and streptophyte counterparts, the *N. olivacea*, *Pyramimonas* and the two mamiellalean cpDNAs possess two identical copies of a large inverted repeat (IR) that are separated by single copy (SC) regions. The quadripartite architecture of the *N. olivacea* and *Pyramimonas* cpDNAs is ancestral (i.e. the SC regions are of vastly unequal size, each containing the highly conserved set of genes typically found in streptophyte cpDNAs, and the rRNA operon encoded by the IR is transcribed toward the small SC region), whereas the gene partitioning pattern observed for the two mamiellalean algae is highly derived.

Analysis of 70 concatenated chloroplast genes and encoded proteins from 24 taxa provided new insights into prasinophyte evolution even though the relationships among all prasinophyte clades could not be explored [25]. The inferred phylogenies disclosed a sister relationship between the Mamiellophyceae and Pyramimonadales and also offered compelling evidence that the green algal partner in the secondary endosymbiosis that gave rise to euglenids was a member of the Pyramimonadales. Consistent with this proposed sister relationship, the Mamiellophyceae and Pyramimonadales form a weakly supported clade in the ML tree reported by Nakayama et al. [29] on the basis of 18S rDNA data; however, these two monophyletic groups are recovered as neighboring lineages in most published 18S rDNA trees. The most important discordance between the chloroplast and 18S rDNA trees concerns the position of *Nephroselmis*: the branch occupied by this genus represents the earliest divergence in chloroplast trees but is part of a later-diverging group of prasinophytes (clades III, V and VII) in 18S rDNA trees. Remarkable differences in the branching order of prasinophyte lineages were also observed between 18S rDNA trees and a phylogeny of 47 green plants inferred from complete cpDNA-encoded rRNA operon sequences lineages [15]. The nodes separating most major prasinophyte lineages in the latter chloroplast phylogeny received weak levels of support.

In the present investigation, we have sequenced the chloroplast genomes of five picoplanktonic prasinophytes belonging to two clades that have not been previously sampled for chloroplast genome analysis, clades VI (Prasinococcales) and clade VII. We have also examined the cpDNA of *Nephroselmis astigmatica*, a clade-III member representing a lineage distinct from *Nephroselmis olivacea* (see [15, 29]). These genome sequences were compared with those of previously examined prasinophytes in order to gain more information on the relationships among prasinophyte lineages and on chloroplast genome evolution. Our phylogenomic analyses revealed that the branching order observed for the six examined prasinophyte clades is largely congruent with previously reported 18S rDNA trees [12, 15] but in contrast to the latter trees, most internal nodes received robust support. The genomes of all the newly investigated picoplanktonic prasinophytes are economically packed and substantially smaller than those observed for prasinophytes with larger cell size, further confirming the notion that small cells have small genomes. Unexpectedly, we found that the three picoplanktonic species occupying the deepest branch (i.e. belonging to the Prasinococcales) harbor several chloroplast genes not previously documented for chlorophytes.

## Results and discussion

**Figure 1:**
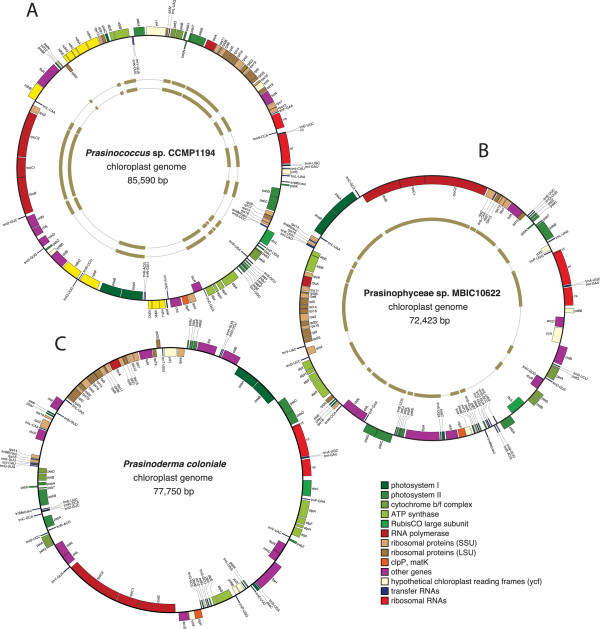
**Gene maps of prasinococcalean chloroplast genomes. (A)** *Prasinococcus*, **(B)** Prasinophyceae *sp.* MBIC 10622 and **(C)** *Prasinoderma*. Filled boxes represent genes, with colors denoting gene categories as indicated in the legend at the bottom the figure. Genes on the outside of each map are transcribed counterclockwise; those on the inside are transcribed clockwise. On panels **A** and **B**, thick lines in the inner rings denote the gene clusters that were found to be conserved in pair-wise comparisons of the three clade-VI genomes. In panel **A**, the inner rings from the inside to the outside indicate the levels of synteny between the *Prasinococcus* cpDNA and those of Prasinophyceae sp. and *Prasinoderma*, respectively, whereas the level of synteny between the latter two genomes is shown in panel **B**.

**Figure 2:**
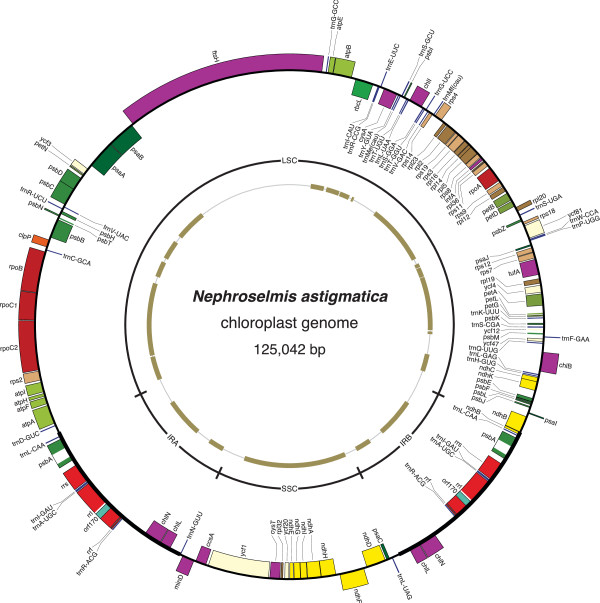
**Gene map of the** ***Nephroselmis astigmatica*** **choroplast genome.** Filled boxes represent genes, with colors denoting categories as indicated in Figure 1. Genes on the outside of the map are transcribed counterclockwise; those on the inside are transcribed clockwise. The outermost inner ring indicates the positions of the IR and SC regions. Thick lines in the innermost ring represent the conserved gene clusters between *Nephroselmis astigmatica* and *Nephroselmis olivacea* cpDNAs.

**Figure 3:**
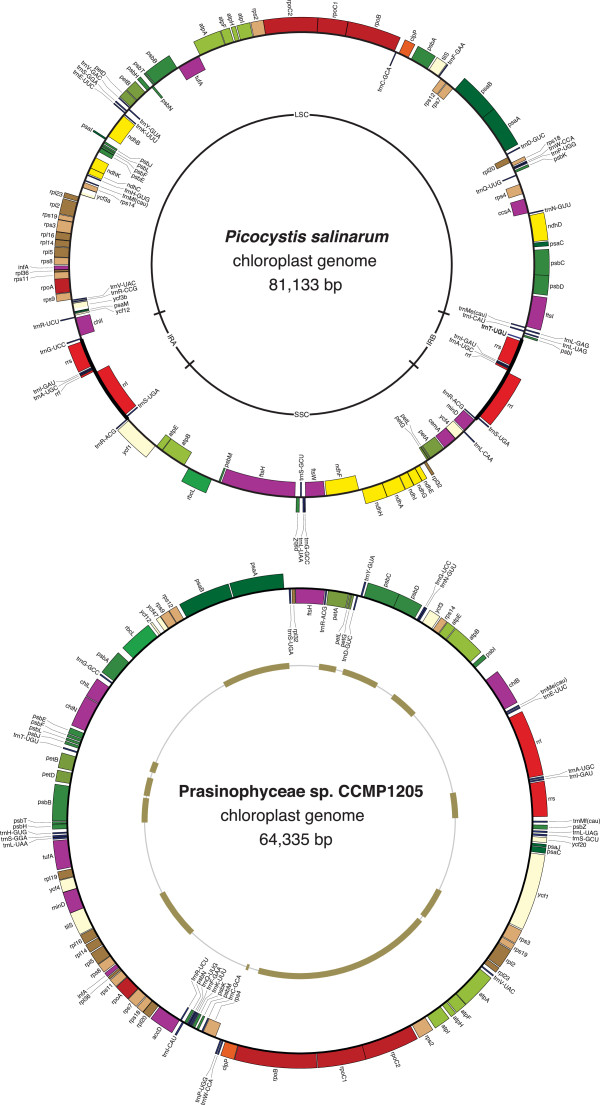
**Gene maps of the** ***Picocystis salinarum*** **and Prasinophyceae sp. CCMP 1205 chloroplast genomes.** Filled boxes represent genes, with colors denoting categories as indicated in Figure 1. Genes on the outside of each map are transcribed counterclockwise; those on the inside are transcribed clockwise. The intron sequences bordering the *Picocystis ycf3* exons (*ycf3*a and *ycf3*b) are spliced in *trans* at the RNA level. The IR and SC regions of the *Picocystis* genome are represented on the inner ring. The gene clusters shared by the *Picocystis* and Prasinophyceae sp. CCMP 1205 genomes are displayed on the ring inside the gene map of the latter genome.

**Figure 4:**
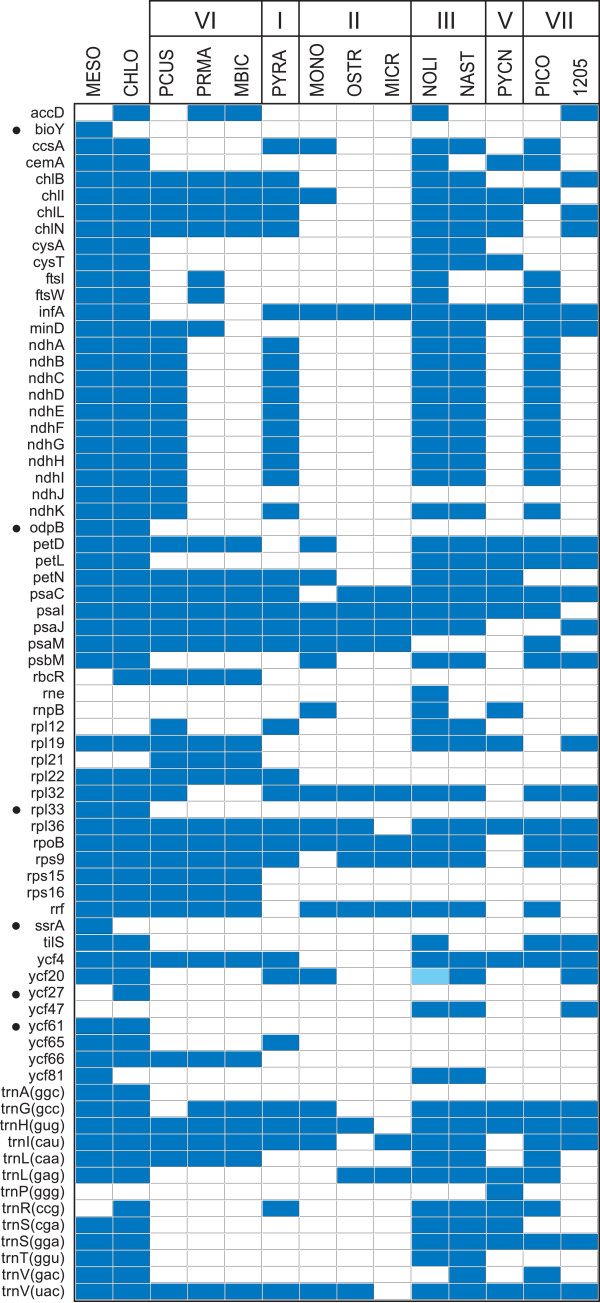
**Differences between the chloroplast gene repertoires displayed by prasinophytes and the deep-branching streptophytes** ***Mesostigma viride*** **and** ***Chlorokybus atmophyticus*** **.** The conserved genes missing in one or more prasinophyte genome as well as the six conserved genes found in *Mesostigma* and/or *Chlorokybus* but absent from all prasinophytes are indicated in the figure; the streptophyte-specific genes are denoted by filled circles. The presence of a gene is indicated by a dark blue box and the presence of a pseudogene by a light blue box. Species names are abbreviated as in Table 1. Although *rpl36*, *trnH* (gug) and *trnV* (uac)) are recorded as missing in *Micromonas,* all three genes are probably present because three lines of evidence suggest that the genome sequence in the [GenBank:NC_012575] accession is partial and that a missing segment contains these genes: 1) the three genes are conserved in all other compared green algae, 2) given that chloroplast gene order is colinear in *Ostreococcus* and *Micromonas,* they are predicted to be contiguous and located between *psbB* and *trnG (ucc*) 3) these predicted positions correspond to the circularization endpoints of the genome assembly deposited in [GenBank:NC_012575]. Species names for prasinophytes are abbreviated as in Table 1. A total of 75 genes are shared by all compared prasinophyte cpDNAs: *atpA, B, E, F, H, I, clpP, ftsH, petA, B, G, psaA, B, psbA, B, C, D, E, F, H, I, J, K, L, N, T, Z, rbcL, rpl2, 5, 14, 16, 20, 23, rpoA, C1, C2, rps2, 3, 4, 7, 8, 11, 12, 14, 18, 19, rrl, rrs, tufA, ycf1, 3, 12, trnA* (ugc)*, C* (gca)*, D* (guc)*, E* (uuc)*, F* (gaa)*, G* (ucc)*, I* (gau)*, K* (uuu)*, L* (uaa)*, L* (uag)*, Me* (cau)*, Mf* (cau)*, N* (guu)*, P* (ugg)*, Q* (uug)*, R* (acg)*, R* (ucu), *S* (gcu)*, S* (uga)*, T* (ugu)*, W* (cca) and *Y* (gua).

**Figure 5:**
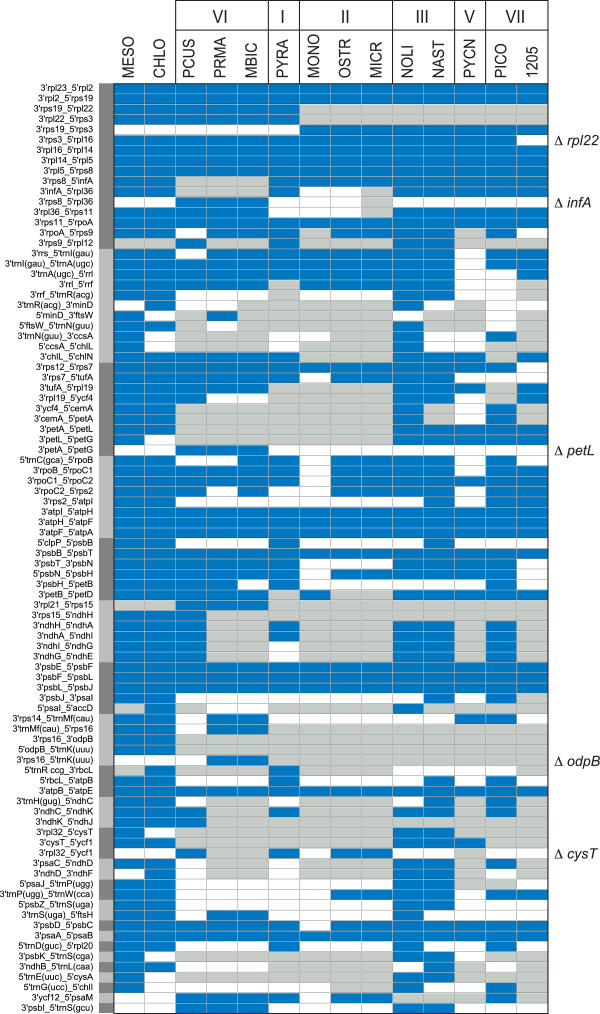
**Distribution of ancestral gene pairs among the 12 prasinophyte cpDNAs examined in this study.** We selected all the gene pairs that are shared by at least two prasinophytes from distinct lineages and also by one or both of the streptophytes *Mesostigma* and *Chlorokybus*. In addition, when one of the genes in a given gene pair was missing from several prasinophyte lineages or from the two streptophytes, gene pairs conserved in a single prasinophyte lineage or missing from streptophytes were selected. The presence of a gene pair is denoted by a dark blue box; a gray box indicates that at least one gene is missing due to gene loss. The gene pairs forming larger conserved clusters are grouped and individual genes that were cleanly deleted from some of these clusters are indicated on the right of the figure. Species names are abbreviated as in Table 1.

The six prasinophyte chloroplast genomes sequenced in the course of this study differ extensively from one another at several levels. Their gene maps are shown in Figures 1, 2 and 3 and their structural features are compared with those previously observed for six other prasinophytes in Table 1. The newly investigated cpDNAs of picoplanktonic species belonging to clades VI and VII vary in size from 64,335 (in Prasinophyceae sp. CCMP 1205, clade VII) to 85,590 bp (in *Prasinococcus,* clade VI), encode between 100 (in Prasinophyceae sp. CCMP 1205) and 115 genes (in *Prasinococcus*) that are densely packed, and with the exception of the *Picocystis* cpDNA (clade VII), contain no IR encoding the rRNA operon. The representative of clade III, *Nephroselmis astigmatica*, exhibits a larger and more gene-rich genome that encodes 123 genes and displays an ancestral quadripartite structure. At the level of gene content, important differences were found within and between clades (Figure 4). Although pairwise genome comparisons revealed that numerous genes form conserved clusters (Figures 1, 2 and 3), gene order is substantially scrambled in all three sampled lineages and multiple ancestral clusters are disrupted in clades VI and VII (Figure 5).

**Table 1:** **General features of the prasinophyte cpDNAs compared in this study**

Taxon		Size				Introns ^b^			
Name	Label	Total (bp)	IR (bp)	A + T (%)	Genes ^a^	GI	GII	Intergenic ^c^(%)	Repeats ^d^(%)
**Clade VI**									
*Prasinococcus* sp. CCMP 1194	PCUS	85,590		67.9	115			14.3	0.9
Prasinophyceae sp. MBIC 106222	MBIC	72,423		62.1	103			13.4	1.1
*Prasinoderma coloniale* CCMP 1220	PRMA	77,750		65.9	106			16.0	0.4
**Clade I**									
*Pyramimonas parkeae* CCMP 726	PYRA	101,605	13,057	65.3	110		1	22.4	0.5
**Clade II**									
*Micromonas* sp. RCC 299	MICR	72,585	7,307	61.2	86^e^			19.0	0
*Monomastix* sp. OKE-1	MONO	114,528		61.0	94	5	1	44.6	16.9
*Ostreococcus tauri*	OSTR	71,666	6,824	60.1	88		1	15.1	0
**Clade III**									
*Nephroselmis astigmatica* NIES 252	NAST	125,042	13,742	59.5	123	2		18.6	0.3
*Nephroselmis olivacea* NIES 484	NOLI	200,801	46,137	57.9	128^f^			45.6	0.5
**Clade V**									
*Pycnococcus provasolii* CCMP 1203	PYCN	80,211		60.5	99^g^		1	14.0	0
**Clade VII**									
*Picocystis salinarum* CCMP 1897	PICO	81,133	10,364	62.7	114		1	9.2	0
Prasinophyceae sp. CCMP 1205	1205	64,335		63.3	100			10.0	0

^a^Duplicated genes were counted only once.

^b^Number of group I (GI) and group II (GII) introns is given.

^c^Only the ORFs coding for proteins of known functions or having recognized domains were considered as genes.

^d^Nonoverlapping repeat elements were mapped on each genome with RepeatMasker [70] using the repeats ≥30 bp identified with REPuter [69] as input sequences.

^e^This value is probably an underestimate because the genome sequence appears to be incomplete and missing three genes (see the legend of Figure 4).

^f^The *ycf20* pseudogene, which corresponds to the annotated *orf111*, was not counted.

^g^This value differs from that reported previously [25] because an additional gene, *rrf* (coordinates 33313–33429 in [GenBank:NC_012097]), was identified using RNAmmer [64] in the course of this study.

Because an accurate phylogenetic framework is essential to track the suite of cpDNA changes that took place during prasinophyte evolution, we will present our phylogenomic analyses of concatenated cpDNA-encoded proteins before elaborating further on the structural features of the newly sequenced prasinophyte genomes.

### The phylogenies inferred from cpDNA-encoded proteins are essentially congruent with the branching order of prasinophyte clades in 18S rDNA trees

**Figure 6:**
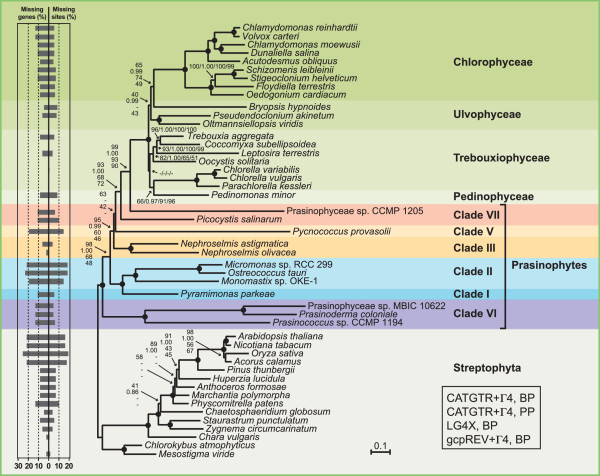
**Relationships among prasinophyte lineages inferred using a data set of 14,382 positions assembled from 71 cpDNA-encoded proteins of 47 green plant taxa.** Trees were inferred using PhyloBayes under the CATGTR + Γ4 model and RAxML under the LG4X and gcpREV + Γ4 models. In the ML analyses, the data set was partitioned by gene, with the model applied to each of the 71 partitions. The Bayesian majority-rule consensus tree is presented. Support values are reported on the nodes: from top to bottom, or from left to right, are shown the BP values for the CATGTR + Γ4 analyses, the PP values for the CATGTR + Γ4 analyses, and the BP values for the LG4X and gcpREV + Γ4 analyses. Dashes (−) indicate lower than 0.95 PP or 40% BP support values; black dots indicate that the corresponding nodes received 1.00 PP and 100% BP support values. The histograms on the left indicate the proportion of missing genes and missing sites for each taxon. The scale bar denotes the estimated number of amino acid substitutions per site.

A data set of 14,382 unambiguously aligned positions was assembled from 71 cpDNA-encoded proteins of 32 chlorophytes and 15 streptophytes, the streptophyte sequences being used as outgroup. Amino acid sequences rather than nucleotide sequences were chosen for our phylogenomic analyses because, in analyses of ancient divergences, amino acid data sets are less prone than nucleotide data sets to saturation problems, convergent compositional biases and convergent codon-usage biases [30–32]. The amino acid data set of 14,382 positions was first analyzed using PhyloBayes and the site-heterogeneous CATGTR + Γ4 model of amino acid substitutions. This model is known to provide a better fit than site-homogeneous models, thus minimizing the impact of systematic errors stemming from the difficulties to detect and interpret multiple substitutions [33–36]. The majority-rule consensus tree resolved six independent lineages for the 12 sampled prasinophytes (Figure 6). We observed with high bootstrap proportion (BP) and posterior probability (PP) support that the three picoplanktonic species belonging to clade VI (i.e. the Prasinococcales) occupies the earliest branch of the Chlorophyta and that the representatives of clades I and II (Pyramimonadales + Mamiellophyceae) form a clade representing the second deepest divergence. As expected, the two *Nephroselmis* species are allied (clade III), forming the third deepest branch of the tree, a branch also robustly supported. The next divergence is represented by the clade-V member, *Pycnococcus*; the relative position of this lineage is uncertain as it received the lowest BP support (only 63%). The two remaining prasinophytes, *Picocystis* and Prasinophyceae sp. CCMP 1205, which have both been assigned to clade VII, form separate and robustly supported lineages, with the latest diverging lineage being occupied by Prasinophyceae sp. CCMP 1205. The latter lineage is sister to a 100% BP supported clade containing the Ulvophyceae, Trebouxiophyceae and Chlorophyceae (UTC clade) and of these classes, only the Chlorophyceae is resolved as a strongly supported monophyletic group. Even though the branching order of lineages within the UTC clade is in agreement with a previous chloroplast phylogenomic study [37], this topology might not reflect the true organismal relationships as taxon sampling is sparse (see [38]).

**Figure 7:**
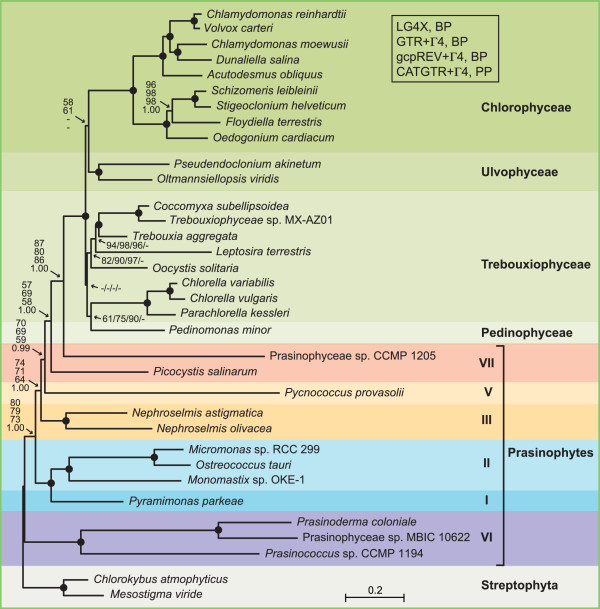
**Relationships among prasinophyte lineages inferred using a data set of 15,549 positions assembled from 79 cpDNA-encoded proteins of 34 green plant taxa.** Trees were inferred using PhyloBayes under the CATGTR + Γ4 model and RAxML under the LG4X, GTR + Γ4 and gcpREV + Γ4 models. In the ML analyses, the data set was partitioned by gene, with the model applied to each of the 79 partitions. The Bayesian majority-rule consensus tree is presented. Support values are reported on the nodes: from top to bottom, or from left to right, are shown the BP values for the LG4X, GTR + Γ4 and gcpREV + Γ4 analyses, and the PP values for the CATGTR + Γ4 analyses. Dashes (−) indicate lower than 0.95 PP or 40% BP support values; black dots indicate that the corresponding nodes received 1.00 PP and 100% BP support values. The scale bar denotes the estimated number of amino acid substitutions per site.

The amino acid data set of 14,382 positions was also analyzed using RAxML under the gcpREV + Γ4 and LG4X models, with each model applied to the data set partitioned by gene. LG4X is a mixture model based on four substitution matrices [39], whereas gcpREV is an empirical model derived from green plant chloroplast sequences [40]. The recovered trees were found to be essentially congruent with the Bayesian tree obtained under CATGTR + Γ4; however, the interrelationships between the prasinophyte lineages were weakly to moderately supported (Figure 6). Indeed, all prasinophyte lineages, except the Prasinophyceae sp. CCMP 1205, received BP support lower than 72%. Given this result, we asked whether ML trees inferred from an amino acid data set with a larger number of characters (15,549 positions) could provide better support for the relationships among prasinophyte lineages. This second data set was assembled from 79 cpDNA-encoded proteins of 32 chlorophytes and two streptophytes, and trees were inferred using PyloBayes under the CATGTR + Γ4 model and RAxML under GTR + Γ4, LG4X, and gcpREV + Γ4 (Figure 7). In all phylogenies, the branching order of prasinophyte lineages was identical to that shown in Figure 6, and higher BP support values were observed for the positions of the early-diverging prasinophytes lineages (clade VI, clades I + II and clade III) as compared to the ML analyses of the original amino acid data set.

Systematic errors are common in phylogenetic studies not only when taxon sampling is sparse but also when some of the taxa produce long branches [36]; indeed, these long branches tend to associate erroneously with those of other taxa showing high sequence divergence, yielding the long-branch artifact [41]. In this context, we note that three of the four prasinophyte lineages occupied by picoplanktonic species, i.e. Prasinococcales, *Pycnococcus* and Prasinophyceae sp. CCMP 1205, display longer branches than the other prasinophyte lineages. It is unlikely, however, that the branching order reported here for prasinophyte lineages was hampered by the long-branch attraction phenomenon because the phylogenies we inferred using various models, including the heterogeneous model of evolution CATGTR + Γ4, are essentially identical and that their topology is congruent with the branching order of major prasinophyte lineages in nuclear-encoded SSU trees [12, 15, 29]. Furthermore, it seems improbable that the prasinophyte relationships resolved in our study were affected by convergent compositional biases and convergent codon-usage biases because we found that the reported amino acid-based phylogenies are congruent with the RAxML tree inferred from a fully degenerated nucleotide data set obtained by substituting the nucleotides with ambiguity codes that allow for all possible synonymous changes (data not shown).

Our chloroplast phylogenomic trees differ from phylogenies based on 18S rDNA data by the sister relationship identified for the Pyramimonadales and Mamiellophyceae [12, 15], the positioning of the Pycnococcaceae after the emergence of the Nephroselmidophyceae [12, 15, 29] and the finding that the two representatives of clade VII are not allied [12, 15, 29]. In the 18S rDNA trees reported by Guillou et al. [12] and Marin and Melkonian [15], the Pyramimondales diverge before the Mamiellophyceae but support for this earlier divergence is very low. Considering that the branch leading to *Pycnococcus* (Pycnococcaceae) received weak support in both the chloroplast and 18S rDNA trees, the differences related to the relative position of this lineage are also not unexpected. Note here that 18S rDNA trees either resolve the Pycnococcaceae as an earlier divergence compared to the Nephroselmidophyceae [15, 29] or group these two lineages in a poorly supported clade [12]. Likewise, in light of the weak support observed for clade VII in 18S rDNA trees as well as in the analysis of the chloroplast rDNA operon by Marin and Melkonian [15], resolution of *Picocystis* (clade VIIC in [12]) and Prasinophyceae sp. CCMP 1205 (clade VIIA in [12]) as neighboring lineages in our phylogenomic analyses does not necessarily represent an inconsistency.

Considering that the prasinophyte clades IV, VIII and IX were not sampled in our study, a number of other issues could not be addressed concerning the interrelationships between the major prasinophyte clades. Representatives of clade IV (Chlorodendrophyceae) would be expected to affiliate to the core chlorophytes (i.e. the clade containing the Pedinophyceae, Trebouxiophyceae, Ulvophyceae and Chlorophyceae), although the exact position of clade IV within this group is still unclear. Concerning the clades VIII and IX, which were defined uniquely by environmental 18S rDNA sequences, sampling of these groups will await the availability of algal cultures in public collections. Because these clades appear to be loosely affiliated with the Nephroselmidophyceae and Pycnococcaceae, respectively [14], analyses of chloroplast genomes from representative species would be very helpful to resolve the position of the Pycnococcaceae.

### The small, IR-less cpDNAs of the clade-VI picoalgae (Prasinococcales) feature six genes not previously documented in chlorophytes

The small sizes of the three examined prasinococcalean chloroplast genomes (72.4 to 85.6 kb) are accounted for by losses of genes and of the IR, tight packaging of the retained genes and the absence of introns (Table 1 and Figure 4). With 103 to 115 genes, *Prasinococcus*, *Prasinoderma*, and Prasinophyceae sp. MBIC 106222 have lost numerous genes compared to *Nephroselmis olivacea* (128 genes) but not as many as the tiny prasinophytes from clades II and V (86 to 98 genes). Most importantly, these prasinococcaleans have retained six genes that have not been previously identified in other chlorophytes: *ndhJ*, *rbcR*, *rpl21*, *rps15*, *rps16*, and *ycf66* (Figure 4). Some of these picoalgae also feature genes that were reported only in the chloroplast of *Nephroselmis olivacea* (*ftsI* and *ftsW*) or *Pyramimonas* (*rpl22)*. Of the three prasinococcaleans examined, the earliest-diverging taxon (i.e. *Prasinococcus,* see Figure 6) displays the largest gene repertoire. Its 115 genes include the genes encoding all 11 subunits homologous to the mitochondrial NADH:ubiquinone oxidoreductase (*ndh* genes), whereas the cpDNAs of *Prasinoderma* and Prasinophyceae sp. MBIC 10622 are entirely lacking these genes. Furthermore, it is worth mentioning that the *trnE* (uuc) gene coding for glutamyl-tRNA, which is a crucial RNA component not only for protein synthesis but also for chlorophyll synthesis [42], is duplicated in the chloroplasts of *Prasinoderma* and Prasinophyceae sp. MBIC 10622. Duplication of *trnE* (uuc) also occurred prior to the divergence of the Chlamydomonadales and Sphaeropleales (Chlorophyceae) [19, 43]; however, whether the acquisition of the extra gene copy conferred a biological advantage is still unknown. Note that another tRNA gene, *trnL* (uag), is duplicated in Prasinophyceae sp. MBIC 10622.

The set of six chloroplast genes found in the Prasinococcales but missing in all other chlorophyte clades provides independent support for the ancestral status of the Prasinococcales within the prasinophyte lineages. This is because positioning of the Prasinococcales as the first branch of the Chlorophyta, rather than as a later divergence, yields the most parsimonious scenario of losses for these six genes, all of which, except *ndhJ*, are predicted to have been lost only once. According to this scenario, *ndhJ* sustained loss not only just after the emergence of the Prasinococcales but also in the lineage leading to the Prasinophyceae sp. MBIC 10622.

In terms of gene order, the *Prasinoderma* and Prasinophyceae sp. MBIC 10622 cpDNAs more closely resemble each other (the 19 shared gene clusters include 67% of the *Prasinoderma* and 69% of the Prasinophyceae genes) than do the *Prasinococcus* cpDNA and each of the former genomes (the 17 and 16 shared clusters include 55% of the *Prasinoderma* and 54% of the Prasinophyceae genes) (Figures 1A and B). Most of the chloroplast gene clusters that are usually shared between streptophytes and chlorophytes have been preserved in prasinococcaleans (Figure 5); one notable exception is the rRNA operon which has been fractured between *rrs* (SSU rRNA gene) and *trnI* (cau) in *Prasinococcus*. The newly identified *rps15*, *rps16* and *ndhJ* genes reside in the same gene context as their streptophyte counterparts and some of the missing genes (*infA*, *petL*, *cysT* and *odpB*) appear to have been cleanly excised from ancestral gene clusters.

### The ancestral quadripartite structure of *Nephroselmis astigmatica*cpDNA features an IR three-fold smaller than its *N. olivacea*homolog

Prior to this study, the IR-containing chloroplast genome of *Nephroselmis olivacea* (Nephroselmidophyceae, clade III) was known to be the prasinophyte cpDNA with the most ancestral pattern of evolution. Its main attributes include a quadripartite structure very similar to those of the streptophyte algae *Mesostigma* [44] and *Chlorokybus* [22], a large gene repertoire, and an ancestral gene organization [28]. The impressive size of its IR (46,136 bp), however, represents an unusual feature. Although it carries several additional conserved genes relative to its *Pyramimonas* counterpart [25], which is more than three-fold smaller, two separate regions totaling about 27 kb lack any genes typically found in cpDNAs. Based on analyses of A + T content and base composition of resident ORFs, it was suggested that these IR regions were acquired by lateral transfer [28]; this hypothesis was substantiated by the subsequent discovery that the *orf389* encodes a putative protein with the conserved domain of phage associated DNA primases [25].

Recent 18S rDNA phylogenies have uncovered multiple lineages in the Nephroselmidophyceae [29]. In the present study, sampling of *Nephroselmis astigmatica*, a representative of a lineage distinct from *Nephroselmis olivacea,* enabled us to gain insight into the stability of the quadripartite structure and ancestrally inherited gene clusters of cpDNA in the Nephroselmidophyceae. Compared to its *Nephroselmis olivacea* counterpart, the *Nephroselmis astigmatica* cpDNA has a much reduced size (125,042 bp), which is mostly accounted for by a smaller IR (Table 1). At 13,742 bp, the IR of this species is comparable in size to that observed for *Pyramimonas* but is almost twice as large as that found for the mamiellaleans *Ostreococcus* and *Micromonas*. Although the IRs of the two *Nephroselmis* species differ in gene content in the region upstream of the rRNA operon, some of the genes downstream of this operon are conserved (*trnR* (acg), *chlN*, and *chlL,* see Figure 2). A notable difference concerns the location of *trnL* (caa): this gene lies at the immediate border of the large SC region in the *Nephroselmis astigmatica* IR (Figure 2); in contrast, it occurs near the small SC region in the *Nephroselmis olivacea* IR. Considering that *trnL* (caa) is usually included within the large SC region in other green plant cpDNAs having an ancestral quadripartite structure (e.g. *Mesostigma* [44] and *Chlorokybus* |[22]), the presence of this gene upstream of the rRNA operon in the *Nephroselmis astigmatica* IR can be simply explained by the expansion/contraction of the IR through gene conversion events [45]. With regards to the two SC regions, the gene content of each in both *Nephroselmis* cpDNAs fully conforms to the ancestral gene partitioning pattern. Given the conventional size of the *Nephroselmis astigmatica* IR, the presumably viral sequences in the *Nephroselmis olivacea* IR were likely captured specifically in the lineage containing the latter species. In this lineage, *trnL* (caa) switched position relative to the rRNA operon, probably by two successive inversions of internal IR sequences containing this operon. Positional changes of genes from one side of the rRNA operon to the other appear to have been frequent during chlorophyte evolution, as they have been observed for other chloroplast genomes displaying slight deviations from the ancestral quadripartite structure (e.g. the cpDNA of *Pyramimonas* [25], *Pedinomonas* [46], and the trebouxiophyte *Parachlorella* [46].

The *Nephroselmis astigmatica* chloroplast has a deficit of only five genes relative to that of *Nephroselmis olivacea*: seven genes are missing (*accD*, *cemA*, *ftsI*, *ftsW*, *tilS*, *rne,* and *rnpB*) and two extra ones are present (*ycf20* and *trnV* (gac)) (Figure 4). All of these nine genes display sporadic distributions in the other chlorophytes examined thus far. Interestingly, *trnV* (gac) is reported for the first time in chlorophyte cpDNAs; it is encoded not only in the *Nephroselmis astigmatica* cpDNA but also in the *Picocystis* (clade VII) genome.

The two *Nephroselmis* genomes share 18 blocks of colinear sequences that include a total of 88 genes (i.e. 72% of the *Nephroselmis astigmatica* and 69% of the *Nephroselmis olivacea* genes) (Figure 2). The largest conserved clusters, which extend from *rpl23* to *petD* and from *trnW* (cca) to *ycf12,* contain 16 and 15 genes, respectively. The *Nephroselmis astigmatica* genome contains several ancestral gene linkages that are missing in *Nephroselmis olivacea* and the Prasinococcales (e.g. 5′*clpP*-5′*psbB*, 3′*psaI*-3′*psbJ*, 3′*rps2*-5′*atpI*, 5′*rbcL*-5′*atpB*, 3′*trnH* (gug)-5′*ndhC*, see Figure 5). Conversely, we find ancestral gene clusters in *Nephroselmis olivacea* that are missing from *Nephroselmis astigmatica* and prasinococcalean species (e.g., 3′*trnS* (uga)-5′*ftsH*, 5′*rpl20*-5′*trnD* (guc)); however, they are less abundant, indicating that ancestral gene linkages have undergone a higher degree of erosion in the *Nephroselmis olivacea* lineage.

While *Nephroselmis olivacea* is lacking any intron in its chloroplast, *Nephroselmis astigmatica* harbors two group I introns. These introns are found in the same genes as the five group I introns in *Monomastix* cpDNA (i.e. the large subunit (LSU) rRNA gene or *rrl* and *psbA*). The *Nephroselmis astigmatica rrl* intron is inserted at site 1951 relative to the *Escherichia coli* 23S rRNA and like its *Monomastix* counterpart at the same site, it encodes a putative LAGLIDADG homing endonuclease gene (*orf170*). On the other hand, the *Nephroselmis astigmatica psbA* intron lacks an ORF and occupies an insertion site (site 750 in the corresponding *Mesostigma psbA gene*) that has not been previously reported for any green plant organelle intron.

### The cpDNAs of the two clade-VII representatives display contrasting patterns of evolution

Because the picoalgae *Picocystis* and Prasinophyceae sp. CCMP 1205 are allied in 18S rDNA trees [12, 15, 29], their chloroplast genomes were expected to exhibit more similarity to each other than to their homologs in other prasinophyte clades. Even though they are both very densely packed with genes, they greatly diverge at the levels of their overall structure, gene content and gene order (Table 1). At 64,335 bp, the Prasinophyceae sp. CCMP 1205 cpDNA is the smallest chloroplast genome yet reported for a photosynthetic green plant. It lacks an IR and its 100 genes display a strong bias in distribution between the two DNA strands: two long segments containing 41 and 42 genes, all encoded on the same DNA strand, are separated from another by two smaller stretches of genes encoded on the opposite strand (Figure 3).

In contrast, the 81,133-bp *Picocystis* genome is more gene-rich (114 genes), contains a 10,364-bp IR, and does not exhibit any pronounced strand bias in gene distribution (Figure 3). As revealed by the gene contents of the SC regions, the quadripartite structure of this genome deviates considerably from both the ancestral gene partitioning pattern observed for the *Nephroselmis* cpDNAs and the derived quadripartite structure of the *Ostreococcus* and *Micromonas* genomes [26, 27]. The gene repertoire of the *Picocystis* chloroplast, which displays 22 genes that are missing from its homolog in Prasinophyceae CCMP 1205, includes ten *ndh* genes as well as three genes that have been identified thus far only in members of the Nephroselmidophyceae and/or Prasinococcales (i.e. *ftsI*, *ftsW*, and *trnV* (gac)) (Figure 4).

The *Picocystis* and Prasinophyceae CCMP 1205 cpDNAs show a low level of synteny. Twelve blocks of colinear sequences containing 42 genes (i.e. 36% of the *Picocystis* and 41% of the Prasinophyceae genes) are conserved between these genomes (Figure 3); they consist uniquely of ancestral gene clusters that are present in most streptophyte and chlorophyte genomes. None of the two picoalgae exhibits an intact rRNA operon: even though the genes making up this operon are located in the *Picocystis* IR, the *rrl* and *rrf* (encoding 5S rRNA) genes have been rearranged, and like its *Pyramimonas* homolog, the *rrf* gene of Prasinophyceae sp. CCMP 1205 appears to be missing (Figure 5). Of the two clade-VII cpDNAs, that of *Picocystis* clearly displays the highest degree of ancestral characters at the gene order level and in that regard, it also surpasses its prasinococcalean counterparts (Figure 5).

**Figure 8:**
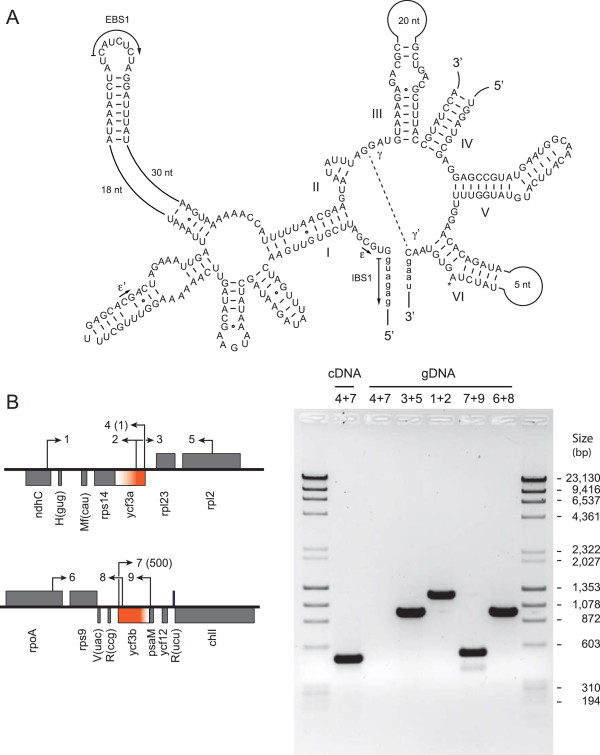
**Analysis of the** ***trans*** **-spliced group II intron in** ***Picocystis ycf3*** **. (A)** Potential intron secondary structure modeled according to Michel et al. (1989) [66]. Exon sequences are shown in lowercase letters. Roman numbers specify the six major structural domains. Tertiary interactions are denoted by dashed lines, arrows, or Greek letterings. EBS and IBS are exon-binding and intron-binding sites, respectively. The asterisk indicates the site of lariat formation. Note that the precise position of the breakpoint within domain IV is unknown. **(B)** Confirmation of intron *trans*-splicing by RT-PCR analysis. The diagrams on the left display the genomic configuration of the *Picocystis ycf3* exons (solid color), with the *trans*-spliced intron sequences shown as color gradients. Primer locations are indicated by numbered arrows (see Methods for primer sequences); the numbers in parentheses denote the nucleotide positions corresponding to the 5′ ends of the primers on the *ycf3* coding sequence after intron removal. Coding regions shown above or below the horizontal line are transcribed to the right or to the left, respectively. PCR assays were carried out on cDNA or genomic DNA (gDNA), with the numbers above the gel lanes indicating the combinations of primers used. The amplicon derived from the PCR assay on cDNA is of the size expected if intron *trans*-splicing occurs to produce the *ycf3* RNA. The identity of this amplicon as well as the insertion position of the intron in the *ycf3* gene were confirmed by DNA sequencing. The amplicons derived from the PCR assays on gDNA have the sizes predicted by the genome map.

While no introns are found in the Prasinophyceae sp. CCMP 1205 cpDNA, a *trans*-spliced group II intron occurs in the *Picocystis* genome. The presence of this intron is somewhat puzzling, considering that, as observed for the Prasinophyceae sp. CCMP 1205 lineage, the chloroplast genome has been extensively streamlined in the lineage leading to *Picocystis*. The *Picocystis* intron is inserted within the *ycf3* gene at the same site (site 124 relative to *Mesostigma ycf3*) as one of the *cis*-spliced *ycf3* introns found in charophytes and land plants [47, 48]. It is split into two pieces that are 6.0 kb apart in the genome and occur as separate transcription units (Figure 3). The fragmentation site lies within domain IV of the potential intron secondary structure (Figure 8A), a common feature of *trans*-spliced group II introns [49]. Like most *trans*-spliced introns of this group, the *Picocystis ycf3* intron lacks an ORF. Given that its domain I is degenerate and that the two pieces flanked by exon sequences must be assembled in *trans* at the RNA level through base-pairing interactions to yield a catalytically active intron structure, one or more nuclear-encoded factors are probably required to facilitate splicing. We have provided experimental evidence by RT-PCR analysis that the *Picocystis* intron is spliced properly and that the *ycf3* gene sequence is continuous at the RNA level (Figure 8b).

Among the Viridiplantae, chloroplast *trans*-spliced group II introns have been identified in streptophytes (in *rps12*), including charophytes [47, 48, 50], and in representatives of all five major evolutionary lineages of the Chlorophyceae (in *psaA*, *psaC*, *petD* and/or *rbcL*) [19, 46, 51–53]. *Trans*-splicing of the tripartite group II intron in the *psaA* gene of the chlorophycean green alga *Chlamydomonas reinhardtii* (Chlamydomonadales) has been shown to be dependent upon at least 14 nuclear-encoded factors [54]. Our finding of a *trans*-spliced intron in a prasinophyte organelle is not unprecedented, as two *trans*-spliced group I introns have been uncovered in the mitochondria of *Prasinoderma* [55]. Both bipartite introns of this tiny prasinophyte occur within the LSU rRNA gene at positions where *cis*-spliced relatives containing homing endonucleases genes are found in organelles of other algae and as observed for other *trans*-spliced group I introns and even *trans*-spliced group II introns, the intron breakpoints correspond to the same region where the ORF occurs in *cis*-spliced orthologs. All *trans*-spliced organelle introns arose undoubtedly from *cis*-spliced orthologs that were fractured by genome rearrangements [56]. It is possible that breakage of the *cis*-spliced ancestor of the *Picocystis ycf3* intron (perhaps an intron gained from streptophytes by horizontal transfer) occurred when the chloroplast genome was less gene-dense and the intron carried a large loop in domain IV, because this would have offered better opportunities for recombination. Subsequently, the *trans*-configuration of the intron would have conferred increased resistance to intron loss by retroprocessing, owing to the lower probability that homologous recombination of a reverse transcribed mature *ycf3* mRNA with the distant exons generates an intronless version of the gene. Therefore, its *trans*-configuration probably explains why it survived in the streamlined chloroplast genome of *Picocystis*.

## Conclusions

The phylogenies we inferred in this study enhance our understanding of the relationships among prasinophyte lineages. They are congruent with the branching order of prasinophyte lineages in nuclear-encoded SSU rDNA trees, even though the two clade-VII members analyzed, *Picocystis* and Prasinophyceae sp. CCMP 1205, form independent lineages instead of a clade, in the chloroplast trees (Figures 6 and 7). Only the position of the Pycnococcaceae relative to the other prasinophyte lineages examined could not be resolved with confidence.

In addition to providing support for the placement of the Prasinococcales in the deepest branch of the Chlorophyta, our comparative analysis of chloroplast genomes underscores the high variability in genome architecture among prasinophyte lineages, highlighting the strong pressure to maintain a small and compact chloroplast genome in picoplanktonic green algae. On the basis of the chloroplast phylogenomic trees we inferred, the IR was lost on at least four occasions during prasinophyte evolution (during the emergence of the Prasinococcales and in the lineages leading to *Monomastix*, *Pycnococcus* and Prasinophyceae sp. CCMP 1205 lineages). Considerable changes in the gene partitioning pattern of the ancestral quadripartite structure took place in two separate picoplanktonic lineages (*Picocystis* and the clade uniting *Ostreococcus* and *Micromonas*). In the Nephroselmidophyceae, the chloroplast genome retained a quadripartite structure of the ancestral type but the IR changed considerably in size and gene content through gain of horizontally transferred sequences (in the lineage leading to *Nephroselmis olivacea*), shifts of IR boundaries, and relocalization of *trnL* (caa) relative to the rRNA operon. Although many genes sustained independent losses in multiple prasinophyte lineages, the Prasinococcales have retained a set of six genes that are missing in all other chlorophytes examined so far. The widely divergent traits uncovered for the genomes of the picoalgae *Picocystis* and Prasinophyceae sp. CCMP 1205 are consistent with the placement of these two clade-VII members in separate lineages. The 64,335-bp IR-less cpDNA of the latter alga is the smallest chloroplast genome yet reported for a photosynthetic green plant. The *ycf3* intron of the tiny *Picocystis* is, to our knowledge, the first chloroplast *trans*-spliced intron documented for prasinophytes; it is also the only intron known in the chloroplast of a picoplanktonic chlorophyte.

## Methods

### Strains and culture conditions

Strains of *Prasinococcus* sp*.* (CCMP 1194), *Prasinoderma coloniale* (CCMP 1220), *Picocystis salinarum* (CCMP 1897) and Prasinophyceae sp. (CCMP 1205) were obtained from the Provasoli-Guillard National Center for Marine Algae and Microbiota [8]. *Nephroselmis astigmatica* (NIES 252) originates from the Microbial Culture Collection at the National Institute of Environmental Studies [9] and Prasinophyceae sp. (NBRC 102842, formally MBIC 106222) from the NITE (National Institute of Technology and Evaluation) Biological Resource Center [57]. All strains were grown in K medium [58] at 18°C under alternating 12 h-light/12 h-dark periods.

### Genome sequencing, assembly and annotation

Total cellular DNA was extracted and run on CsCl-bisbenzimide (1.67 g/ml CsCl, 200 μg/ml bisbenzimide) isopycnic gradients as described [59]. The resulting gradient was fractionated into 40 fractions (120 μl each) using a Density Gradient Fractionation System (Brandel, Gaithersburg, MD, USA). After precipitation with ethanol, DNA from each of the 20 lowest density fractions was digested with EcoRI and the fractions displaying digestion patterns of low complexity DNA on agarose gels were selected for sequencing. However, as the isolation of A + T-rich organellar DNA proved unsuccessful for *Nephroselmis astigmatica,* total cellular DNA of this alga was used for sequencing.

Sequencing of the Prasinophyceae sp. CCMP 1205 cpDNA was carried out using random clones and Sanger chemistry. Random clone libraries were prepared from 1500-2000-bp fragments derived from the A + T rich DNA fractions using the pSMART-HCKan (Lucigen Corporation, Middleton, WI) plasmid. Positive clones were selected by hybridization of each plasmid library with the original DNA used for cloning. DNA templates were amplified using the Illustra TempliPhi Amplification Kit (GE Healthcare, Baie d’Urfé, Canada) and sequenced with the PRISM BigDye terminator cycle sequencing ready reaction kit (Applied Biosystems, Foster City, CA) on Applied Biosystems model 3130XL DNA sequencers, using T3 and T7 primers as well as oligonucleotides complementary to internal regions of the plasmid DNA inserts. The resulting sequences were edited and assembled using SEQUENCHER 5.1 (Gene Codes Corporation, Ann Arbor, MI). Genomic regions not represented in the sequence assemblies or plasmid clones were directly sequenced from polymerase chain reaction (PCR)-amplified fragments using internal primers. Note that the 11 Prasinophyceae CCMP 1205 chloroplast genes that Matsumoto et al. [60] have previously examined share identical or nearly identical sequences with their counterparts in the genome we assembled, thus confirming that this chloroplast genome is that of the genuine CCMP 1205 strain.

For all the other algal cpDNAs, a shotgun library of A + T-rich organellar DNA or total cellular DNA (700 bp fragments) was constructed using the GS-FLX Titanium Rapid Library Preparation Kit from Roche 454 Life Sciences (Branford, CT, USA). Library construction as well as 454 GS-FLX DNA Titanium pyrosequencing were carried out by the Plateforme d’Analyses Génomiques de l’Université Laval [10]. Reads were assembled with gsAssembler 2.5 from the Roche GS Data Analysis Software package, and contigs were visualized, linked, edited and polished using the CONSED 22 package [61]. Contigs of chloroplast origin were identified by BLAST searches against a local database of organelle genomes. Regions spanning gaps in the cpDNA assemblies were amplified by PCR with primers specific to the flanking sequences. Purified PCR products were sequenced using Sanger chemistry with the PRISM BigDye terminator cycle sequencing ready reaction kit (Applied Biosystems, Foster City, CA, USA) by the Plateforme d’Analyses Génomiques. Average genome coverage ranged from 80–290 in the assemblies of all cpDNAs, except that of *Nephroselmis astigmatica*. As an average coverage of less than 10 was obtained for the latter cpDNA, we confirmed the 454 sequence assembly with 6.5 millions paired-end reads of 300 bp generated on the MiSeq sequencing platform (Illumina, San Diego, CA, USA). Following *de novo* assembly of these reads using Ray 2.3.1 [62] and a kmer size of 61, we obtained two overlapping chloroplast contigs, each containing a SC region (small or large SC) flanked at both ends with part of the IR sequence. After identification of the IR-SC junction in each contig, we were able to assemble the two contigs into a circular molecule that has essentially the same sequence as that obtained with the 454 data.

Genes and ORFs were identified on the final assemblies using a custom-built suite of bioinformatics tools as described previously [63]. Genes coding for rRNAs and tRNAs were localized using RNAmmer [64] and tRNAscan-SE [65], respectively. Intron boundaries were determined by modeling intron secondary structures [66, 67] and by comparing intron-containing genes with intronless homologs. Gene maps were drawn using OGDRAW [68].

To estimate the proportion of repeated sequences in each of the sequenced chloroplast genomes*,* repeats ≥ 30 bp were retrieved using REPFIND of the REPuter 2.74 program [69] with the options -f (forward) -p (palindromic) -l (minimum length = 30 bp) -allmax and then were masked on the genome sequence using REPEATMASKER [70] running under the Crossmatch search engine [71].

### RNA extraction and RT-PCR reactions

Total RNA from *Picocystis* was extracted from cells ground in liquid nitrogen with the E.Z.N.A Total RNA Kit of Omega bio-tek (Norcross, GA, USA). To confirm that the *ycf3* transcript undergoes *trans*-splicing and also to verify the insertion position of the *trans*-spliced intron, RT-PCR reactions were performed on the RNA preparation using the Qiagen One-Step RT-PCR kit with the primers ATGCCTAGATCACAACGAAATG (40,810, primer 4) and GTTCGACCTGTTACTTTCAACC (46,919, primer 7). The RT-PCR product was sequenced using Sanger chemistry as described above. The genomic positions of the *ycf3a* and *ycf3b* exons were also confirmed by carrying out PCR reactions on genomic DNA using various combinations of the following primers: CTCATACGTTGTTCGTTGAATG (39,456, primer 1), GAAGCATTCAGTTATTACCGAG (40,711, primer 2), CTCGGTAATAACTGAATGCTTC (40,690, primer 3), TATGTACTTCAGTACCTAGAGG (41,704, primer 5), GCCTATCCGACAAGTCAATTAC (46,061, primer 6), GCAATAGAAAACGATCAGACAG (47,052, primer 8), and TTGCATTACGTTTAGGTACTGC (47,452, primer 9). For each primer, the coordinate of the genome sequence corresponding to the 5′end is indicated in parentheses together with the primer numbering shown in Figure 8B.

### Analyses of gene order data

We used a custom-built program to identify all pairs of signed genes (i.e., taking into account gene polarity) that are conserved in at least two of the 14 compared cpDNAs (those of the 12 prasinophytes and of the streptophyte algae *Mesostigma* and *Chlorokybus*). This program was also employed to detect the regions that display the same gene order in selected pairs of prasinophyte cpDNAs.

### Phylogenomic analyses

The chloroplast genomes of 47 green algae were used for phylogenomic analysis. The GenBank accession numbers of these green algal genomes are as follows: *Mesostigma viride*, [GenBank:NC_002186]; *Chlorokybus atmophyticus*, [GenBank:NC_008822]; *Chara vulgaris*, [GenBank:NC_008097]; *Chaetosphaeridium globosum*, [GenBank:NC_004115]; *Zygnema circumcarinatum*, [GenBank:NC_008117]; *Staurastrum punctulatum*, [GenBank:NC_008116]; *Physcomitrella patens*, [GenBank:NC_005087]; *Marchantia polymorpha*, [GenBank:NC_001319]; *Anthoceros formosae*, [GenBank:NC_004543]; *Huperzia lucidula*, [GenBank:NC_006861]; *Pinus thunbergii*, [GenBank:NC_001631]; *Acorus calamus*, [GenBank:NC_007407]; *Oryza sativa*, [GenBank:NC_001320]; *Nicotiana tabacum*, [GenBank:NC_001879]; *Arabidopsis thaliana*, [GenBank:NC_000932]; *Prasinococcus* sp. CCMP 1194, [GenBank:KJ746597]; *Prasinoderma coloniale* CCMP 1220, [GenBank:KJ746598]; Prasinophyceae sp. MBIC 106222, [GenBank:KJ746602]; *Pyramimonas parkeae*, [GenBank:NC_012099]; *Monomastix* sp. OKE-1, [GenBank:NC_012101]; *Ostreococcus tauri*, [GenBank:NC_008289]; *Micromonas* sp. RCC 299, [GenBank:NC_012575]; *Nephroselmis olivacea*, [GenBank:NC_000927]; *Nephroselmis astigmatica*, [GenBank:KJ746600]; *Pycnococcus provasolii*, [GenBank:NC_012097]; *Picocystis salinarum*, [GenBank:KJ746599]; Prasinophyceae sp. CCMP 1205, [GenBank:KJ746601]; *Pedinomonas minor*, [GenBank:NC_016733]; *Chlorella vulgaris*, [GenBank:NC_001865]; *Chlorella variabilis*, [GenBank:NC_015359]; *Oocystis solitaria*, [GenBank:FJ968739]; *Parachlorella kessleri*, [GenBank:NC_012978]; *Coccomyxa subellipsoidea*, [GenBank:NC_015084]; *Leptosira terrestris*, [GenBank:NC_009681]; *Trebouxia aggregata*, [GenBank:EU123962-EU124002]; *Bryopsis hypnoides*, [GenBank:NC_013359]; *Oltmannsiellopsis viridis*, [GenBank:NC_008099]; *Pseudendoclonium akinetum*, [GenBank:NC_008114]; *Oedogonium cardiacum*, [GenBank:NC_011031]; *Floydiella terrestris*, [GenBank:NC_014346]; *Stigeoclonium helveticum*, [GenBank:NC_008372]; *Schizomeris leibleinii*, [GenBank:NC_015645]; *Acutodesmus obliquus*, [GenBank:NC_008101]; *Chlamydomonas moewusii*, [GenBank:EF587443-EF587503]; *Dunaliella salina*, [GenBank:NC_016732]; *Volvox carteri* f. *nagariensis*, [GenBank:GU084820]; and *Chlamydomonas reinhardtii*, [GenBank:NC_005353].

We selected the protein-coding genes that are shared by at least 25 of the 47 taxa. Seventy-one genes met this criterion: *accD, atpA, B, E, F, H, I, ccsA, cemA, chlB, I, L, N, P, ftsH, infA, petA, B, D, G, L, psaA, B, C, I, J, M, psbA, B, C, D, E, F, H, I, J, K, L, M, N, T, Z, rbcL, rpl2, 5, 14, 16, 20, 23, 32, 36, rpoA, B, C1, C2, rps2, 3, 4, 7, 8, 9, 11, 12, 14, 18, 19, tufA, ycf1, 3, 4, 12.* An amino acid data set of 14,382 positions was prepared as follows: the deduced amino acid sequences from the 71 individual genes were aligned using MUSCLE 3.7 [72], the ambiguously aligned regions in each alignment were removed using TRIMAL 1.3 [73] with the options block = 7, gt = 0.7, st = 0.001 and sw = 3, and the protein alignments were concatenated. Missing characters represent 7.4% of the data set.

The amino acid data set of 14,382 positions was analyzed using both the ML and Bayesian inference methods. The Bayesian phylogeny was inferred using PhyloBayes 3.3f [74] and the site-heterogeneous CATGTR + Γ4 model [34]. To identify the best tree, five independent chains were run for 6,300 cycles under this model and a consensus topology was calculated from the saved trees using the BPCOMP program of PhyloBayes after a burn-in of 1,500 cycles. Under these conditions, the largest discrepancy observed across all bipartitions in the consensus topology (maxdiff) was 0.26, indicating that convergence between the five chains was achieved. To determine the confidence level at each node of the best tree, 100 pseudo-replicates were generated using the SEQBOOT program of the PHYLIP package [75], chains were run using 2,000 cycles (with each cycle sampled) for each pseudo-replicate, and a consensus tree was computed with the READPB program of PhyloBayes after elimination of 500 burn-in trees. A bootstrap consensus tree, whose topology was identical to the best tree, was generated from the 100 resulting consensus trees using the CONSENSE program of the PHYLIP package. The ML analyses were carried out using RAxML 8.0.20 [76] and the LG4X [39] and gcpREV + Γ4 [40] models of sequence evolution. In these analyses, the data set was partitioned by gene, with the model applied to each partition. Confidence of branch points was estimated by fast-bootstrap analysis (f = a) with 100 replicates.

ML and Bayesian phylogenies were also inferred from an amino acid data set of 15,549 positions that was assembled from 79 cpDNA-encoded proteins of 32 chlorophytes and two streptophytes. Relative to the amino acid data set of 14,382 positions, this data set includes the deduced protein sequences of eight extra genes (*cysA, cysT, minD, rpl12, rpl19, tilS, ycf20* and *ycf47*) and the sequences of an additional trebouxiophyte (*Trebouxiophyceae* sp. MX-AZ01, [GenBank:NC_018569]). It was prepared as described above, except that the TRIMAL filtration was carried out with the options block = 6, gt = 0.7, st = 0.005 and sw = 3. Missing characters represent 9.2% of the data set. The RAxML and PhyloBayes analyses were carried out as described for the data set of 14,382 positions.

### Availability of supporting data

The chloroplast genome sequences generated in this study are available in the GenBank database under the accession numbers KJ746597-KJ746602. The data sets supporting the results of this article are available in the TreeBASE repository (Study ID 16332) [77].

## Abbreviations

BP: Bootstrap proportion
cpDNA: Chloroplast DNA
IR: Inverted repeat
LSU: Large subunit
PP: Posterior probability
SC: Single copy
SSU: Small subunit.
